# A 2 × 2 factorial design for the combination therapy of minocycline and remote ischemic perconditioning: efficacy in a preclinical trial in murine thromboembolic stroke model

**DOI:** 10.1186/2040-7378-6-10

**Published:** 2014-10-09

**Authors:** Md Nasrul Hoda, Susan C Fagan, Mohammad B Khan, Kumar Vaibhav, Aizaz Chaudhary, Phillip Wang, Krishnan M Dhandapani, Jennifer L Waller, David C Hess

**Affiliations:** 1 Department of Neurology, Georgia Regents University, Augusta, GA 30912, USA; 2 Department of Medical Laboratory, Imaging and Radiologic Sciences, Georgia Regents University, Augusta, GA 30912, USA; 3 Department of Psychiatry, Georgia Regents University, Augusta, GA 30912, USA; 4 Department of Neurosurgery, Georgia Regents University, Augusta, GA 30912, USA; 5 Department of Biostatistics and Epidemiology, Georgia Regents University, Augusta, GA 30912, USA; 6 Program in Clinical and Experimental Therapeutics, College of Pharmacy, University of Georgia, Augusta, USA; 7 Charlie Norwood VA Medical Centre, Augusta, USA

**Keywords:** Thromboembolic stroke, Minocycline, Remote ischemic perconditioning, Neurovascular protection, Intravenous tPA

## Abstract

**Background:**

After the failure of so many drugs and therapies for acute ischemic stroke, innovative approaches are needed to develop new treatments. One promising strategy is to test combinations of agents in the pre-hospital setting prior to the administration of intravenous tissue plasminogen activator (IV-tPA) and/ or the use of mechanical reperfusion devices in the hospital.

**Methods:**

We performed a 2 × 2 factorial design preclinical trial where we tested minocycline (MINO), remote ischemic perconditioning (RIPerC) and their combination treatment in a thromboembolic clot model of stroke in mice, without IV-tPA or later treated with IV-tPA at 4 hours post-stroke. Cerebral blood flow (CBF) was measured by laser speckle contrast imaging (LSCI), behavioral outcomes as neurological deficit score (NDS) and adhesive tape removal test, and infarct size measurement were performed at 48 hours post-stroke. Mice within the experimental sets were randomized for the different treatments, and all outcome measures were blinded.

**Results:**

RIPerC significantly improved CBF as measured by LSCI in both with and without tPA treated mice (P < 0.001). MINO and RIPerC treatment were effective alone at reducing infarct size (p < 0.0001) and improving short-term functional outcomes (p < 0.001) in the tPA and non-tPA treated animals. The combination treatment of MINO and RIPerC significantly reduced the infarct size greater than either intervention alone (p < 0.05). There were trends in favor of improving functional outcomes after combination treatment of MINO and RIPerC; however combination treatment group was not significantly different than the individual treatments of MINO and RIPerC. There was no “statistical” interaction between minocycline and RIPerC treatments indicating that the effects of RIPerC and MINO were additive and not synergistic on the outcome measures.

**Conclusion:**

In the future, combining these two safe and low cost interventions in the ambulance has the potential to “freeze” the penumbra and improve outcomes in stroke patients. This pre-clinical 2 × 2 design can be easily translated into a pre-hospital clinical trial.

## Introduction

Bold and innovative strategies are needed to break the logjam of failed acute stroke clinical trials. Delivery of drugs and interventions in the ambulance and pre-hospital setting soon after the onset of ischemia is one promising strategy. The Stroke Therapy Academic Industry Roundtable (STAIR) VII concluded that the “strategy of delivering neuroprotective therapies before reperfusion treatments to extend penumbra survival is attractive and could potentiate the benefits of early reperfusion therapy and expand the time window for late reperfusion interventions” [1]. The FAST MAGS clinical trial demonstrated the feasibility of early treatment times (mean 45 minutes) with neuroprotective treatments in the pre-hospital setting and provides the “proof of concept” that interventions can be started in the ambulance in the first hour, the ’golden hour” after the onset of stroke symptom [2,3].

The ideal pre-hospital agent for stroke should have an excellent safety profile, be well tolerated, be easy to administer in the ambulance, be inexpensive, and be safe and potentially beneficial in both ischemic and hemorrhagic stroke [3]. Two interventions that fit this profile are minocycline (MINO) and remote limb ischemic perconditioning (RIPerC). MINO is safe, easy to administer, has a long half-life, and is promising in early phase clinical trials [4]. RIPerC is also safe, well tolerated and has already shown to be feasible with hints of efficacy and activity when administered in the pre-hospital setting in clinical trials of ST segment elevated myocardial infarction (STEMI) and stroke [5,6]. The only published preclinical report in the animal model of intracerebral hemorrhage (ICH) demonstrated that RLIC one hour after ICH does not exacerbate the injury [7]. A recently published clinical trial reported that RLIC in the ambulance was not detrimental in stroke patients with intracerebral hemorrhage (ICH) [6]. Two different phase I clinical trials of the safety and feasibility of RLIC in sub-arachnoid hemorrhage (SAH) concluded that it is well tolerated and safe even in critically ill patients [8,9]. Even after 4-cycles of RLIC, the prothrombin time (PT), and international normalized ratio (INR) remain in the normal range without any hemorrhagic complications [10]. Both MINO and RIPerC work well alone and in combination with intravenous tissue plasminogen activator (IV-tPA) and are ideal adjuvant therapies during reperfusion [11-13]. They both are interventions that “freeze” the penumbra [2].

An innovative approach to stroke would involve using multiple agents in combination in the field prior to administration of IV-tPA in the hospital. In any clinical trial of multiple agents used in combination, the Food and Drug Administration (FDA) would require that each agent be tested alone and in combination [14]. An efficient clinical trial design is be a 2 × 2 factorial design where each agent alone and the combination are tested.

Since IV-tPA is the only FDA-approved drug for stroke, all new agents administered within 3 to 6 hours need to be tested in combination with IV-tPA. Since IV-tPA is effective by clot lysis, the rodent thromboembolic clot model is an ideal model to test promising agents in combination with IV-tPA. Therefore, we conducted a preclinical 2 × 2 design of MINO and RIPerC in a thromboembolic clot model with and without IV-tPA as a foundation for a clinical trial, and to determine if there are any interactions between minocycline and RIPerC.

## Materials and methods

### Animals, experimental groups and procedures

The Institutional Animal Care and Use Committee of Georgia Regents University (GRU) approved all animal procedures. In-house bred 80 C57BL/6 J wild type mid aged male mice kept in GRU’s AAALAC accredited facility (12 ± 1 month old), were used in the following two experiments with and without IV-tPA. In Experiment I, all four groups (n = 10 per group) were subjected to stroke without follow up thrombolysis by IV-tPA. The treatment protocol for the individual and combination therapies was based on a 2 MINO (no vs. yes) by 2 RIPerC (no vs. yes) design as indicated by the different group names in the Table 1a. Minocycline or vehicle was infused at 1 hr post-stroke while RIPerC or its sham procedure was performed at 2 hrs post-stroke, as needed in the relevant groups and explained in the table. In Experiment II, all four groups (N = 10 per group) were subjected to stroke followed by late thrombolysis with IV-tPA at 4 hrs post-stroke. The experimental protocol for the individual and combination treatments were same as in Experiment I (please see Table 1b).

**Table 1:** Study design for the experiment I (without IV-tPA; 1a), and experiment II (with IV-tPA; 1b)

**a, Experiment I: Without IV-tPA post-stroke**		
	**No MINO** (IV-Saline 1 hr post-stroke)	**Yes MINO** (IV-MINO 1 hrs post-stroke)
**No RIPerC** (RIPerC Sham at 2 hrs post-stroke)	Saline, RIPerC Sham (+Veh+Sham Group)	MINO, RIPerC Sham (+MINO Group)
**Yes RIPerC** (RIPerC Therapy at 2 hrs post-stroke)	Saline, RIPerC Therapy (+Veh+RIPerC Group)	MINO, RIPerC Therapy (+MINO+RIPerC Group)
**b, Experiment II: With IV-tPA (or vehicle) at 4 hrs post-stroke**		
	**No MINO** (IV-Saline 1 hr post-stroke)	**Yes MINO** (IV-MINO 1 hrs post-stroke)
**No RIPerC** (RIPerC Sham at 2 hrs post-stroke)	Saline, RIPerC Sham (+Veh+Sham Group)	MINO, RIPerC Sham (+MINO Group)
**Yes RIPerC** (RIPerC Therapy at 2 hrs post-stroke)	Saline, RIPerC Therapy (+Veh+RIPerC Group)	MINO, RIPerC Therapy (+MINO+RIPerC Group)

The sample size estimation, randomization within IV-tPA status (no or yes thrombolysis) and blinding strategies including embolic stroke model and non-invasive RIPerC procedures were similar to as reported earlier by us and in the related supplementary information [12,13,15]. Wherever needed in the groups, MINO was infused intravenously (6 mg/kg bwt) 1 hr post-stroke, and RIPerC therapy was performed at 2 hrs post-stroke with their appropriate sham-operation controls as reported by us [12,13,15]. Instead of laser Doppler flowmetry, laser speckle contrast imaging (LSCI) was used to determine post-stroke cerebral perfusion as discussed below, and percent change in CBF compared to contralateral side was calculated as reported earlier by us [12,13]. All groups were sacrificed 48 hrs post-stroke. Because of higher death rate in aged mice, [15] and anticipated further increase in the mortality due to late IV-tPA treatment, we reduced the size of the clot and used a 7.0 ± 0.5 mm long clot, as reported by us [8]. Neurobehavioral assessments by neurological deficit score (NDS) and sensorimotor function test by adhesive tape test, as well as infarct analyses were performed as recently reported by us in multiple reports [12,13,15].

### Cerebral perfusion by LSCI

LSCI for stroke animals was performed similar to as reported earlier by us for laser Doppler imaging in experimental stroke [12,13], with certain suitable modifications as described below. Mice were anesthetized using isofluorane, body temperature was maintained at 37 ± 0.5°C, the skull was shaved, exposed by a midline skin incision and cleaned. Perfusion images were acquired using PeriCam high resolution LSCI (PSI system, Perimed) with a 70 mW built-in laser diode for illumination and 1388 × 1038 pixels CCD camera installed 10 cm above the skull (speed 19 Hz, and exposure time 6 mSec). Acquired images were analyzed for dynamic changes in CBF using PIMSoft (Perimed). Since anesthesia and stroke procedure affects the cerebral perfusion of uninjured stroke side too, the absolute value from the ipsilateral side was normalized with the value from contralateral side and calculated as percent change as reported by us for laser Doppler imaging [12,13].

### Statistical analyses

All the data are expressed as mean ± SD. Statistical analyses were performed using SAS 9.4 (SAS Institute Inc., Cary, NC). Briefly, a rank transformation was used prior to analysis wherever needed to stabilize variance across groups. A two-factor ANOVA [MINO (no vs. yes) and RIPerC (no vs. yes)] with the interaction within IV-tPA status (no or yes IV-tPA) was used to analyze outcomes. A Tukey’s multiple comparison procedure using the least square means of the MINO × RIPerC interaction term was used to examine post hoc pairwise comparisons. In the absence of a significant interaction, the main effects were considered to be additive when combined. Statistical significance was determined at p < 0.05.

## Results

### Changes in cerebral perfusion by MINO, RIPerC and their combination treatments after embolic stroke with and without IV-tPA

**Figure 1:**
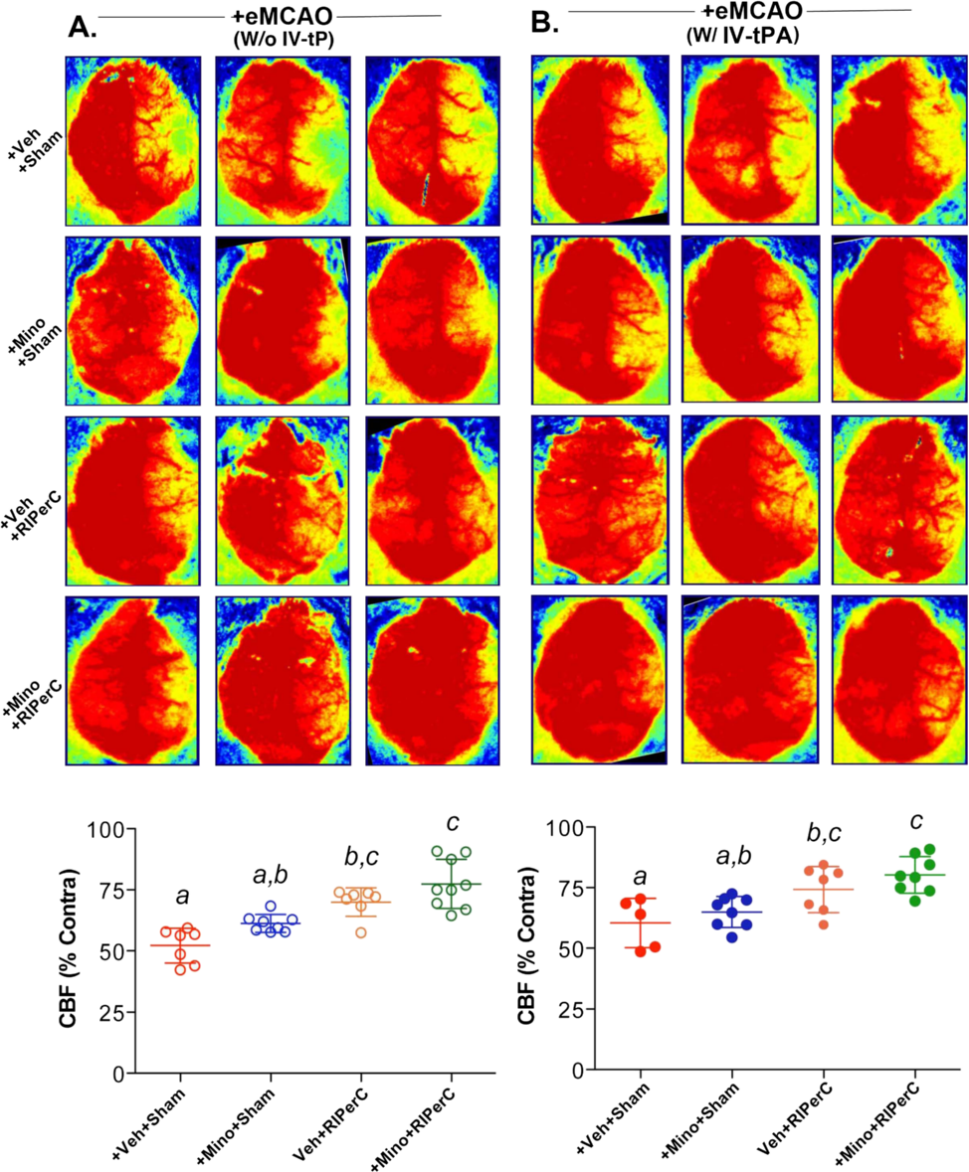
**Cerebral perfusion in the ischemic hemisphere of surviving animals at 48 hrs post-stroke as measured with LSCI and calculated as percent of contralateral value.** Comparisons between the groups were done “within the experimental set” either without or with IV-tPA. Representative ximages from 3 different animals, and plot from the two experimental sets, **A**. Experiment I without IV-tPA; +Veh + Sham (N = 7 survived out of 10; 7/10), +MINO + Sham (N = 8/10), +Veh + RIPerC (N = 7/10) and + MINO + RIPerC (N = 10/10), and **B**. Experiment II with IV-tPA; +Veh + Sham (N = 5 survived out of 10; 5/10), +MINO + Sham (N = 8/10), +Veh + RIPerC (N = 7/10), and + MINO + RIPerC (N = 8/10). For both experiments, data presented as Mean ± SD. Pairs of Means with different letters are significantly different, p < 0.05.

After delivering the partially humanized clot in the ipsilateral side, the overall cerebral perfusion significantly (p < 0.001) dropped as compared to the contralateral side. The changes were ~50% immediately after stroke among the different groups of the two sets of experiments. At 48 hrs post-stroke, there was spontaneous but partial recanalization in the + Veh + Sham group of both sets of experiments (Figure 1A-B). MINO did not improve the CBF significantly with IV-tPA (MINO main effect F_(1,24)_ = 2.76, p = 0.1095) but did without IV-tPA (F_(1,27)_ = 10.01, p = 0.0038), although there was a trend towards increased perfusion at 48 hrs post-stroke possibly due to vascular as well as neuroprotective effects of MINO. In contrast, RIPerC significantly improved the CBF as compared to sham-operated group with (F_(1,24)_ = 20.88, p = 0.0001) or without (F_(1,27)_ = 42.47, p < 0.0001) IV-tPA when assessed at 48 hrs post-stroke. When two individual treatments of MINO at 1 hr followed by RIPerC at 2 hrs post-stroke were used in combination, CBF increased significantly compared to the sham-treated group with (p = 0.0017) or without (p < 0.0001) IV-tPA, as well as compared to MINO alone with IV-tPA (p = 0.0058) or without IV-tPA (p = 0.0005) but not when compared to RIPerC alone with IV-tPA (p = 0.5053) or without IV-tPA (p = 0.1958). This improvement in CBF as a result of combination treatment is possibly due to their additive benefits on vascular protection. In both the sets with (F_(1,27)_ = 0.05, p = 0.8169) and without (F_(1,24)_ = 0.09, p = 0.7624) IV-tPA, there was no interaction between MINO and RIPerC.

### MINO and RIPerC individual treatments, and their combination after embolic stroke conferred neurobehavioral benefits with and without IV-tPA

**Figure 2:**
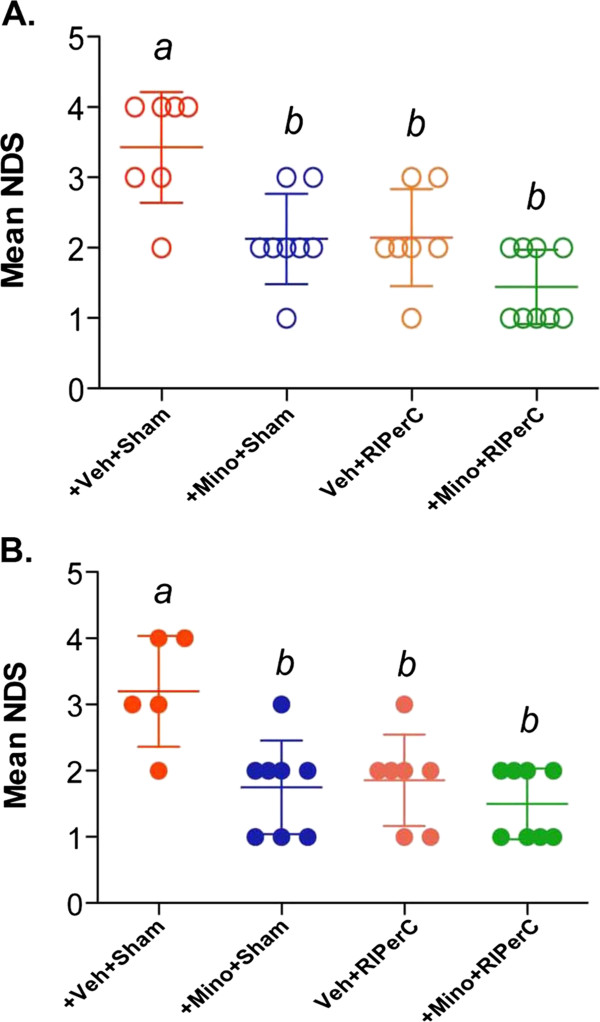
**Assessment of Neurologic Deficit Score (NDS) on Bederson Scale at 48 hours post-stroke. A**. Experiment I without IV-tPA, and **B**. Experiment II with IV-tPA. Data presented as Mean ± SD (N = as above in Figure 1). Pairs of Means with different letters are significantly different, p < 0.05.

Neurobehavioral assessments were performed on the surviving animals from both experiments with and without thrombolysis (Figure 2A-B). In the Experiment I without IV-tPA (no thrombolysis) the interaction between MINO and RIPerC was not statistically significant (F_(1,27)_ = 1.62, p = 0.2134; please see Additional file 1: Figure S1a) but both main effect did show differences (MINO F_(1,27)_ = 17.77, p = 0.0002, RIPerC F_(1,27)_ = 17.14, p = 0.0003). Both individual treatments (MINO only p = 0.0036, RIPerC only p = 0.0056) and combination therapy (p < 0.0001) attenuated the post-stroke NDS significantly as compared to the sham-treatment group without thrombolysis. However, there was no significant difference in the mean NDS of individual treatments vs. combination therapy. In Experiment II with IV-tPA thrombolysis, the interaction between MINO and RIPerC was statistically significant (F_(1,24)_ = 4.33, p = 0.0482; please see Additional file 1: Figure S1b) with the attenuation in the mean NDS in the “no MINO group” from no RIPerC to RIPerC being greater than the attenuation in the “yes MINO group”. All the treatment groups (MINO p = 0.0054, RIPerC p = 0.0128 and their combination p = 0.0011) showed significantly attenuated mean NDS as compared to the sham treatment group but there was no significant difference in the mean NDS among the treatment groups.

**Figure 3:**
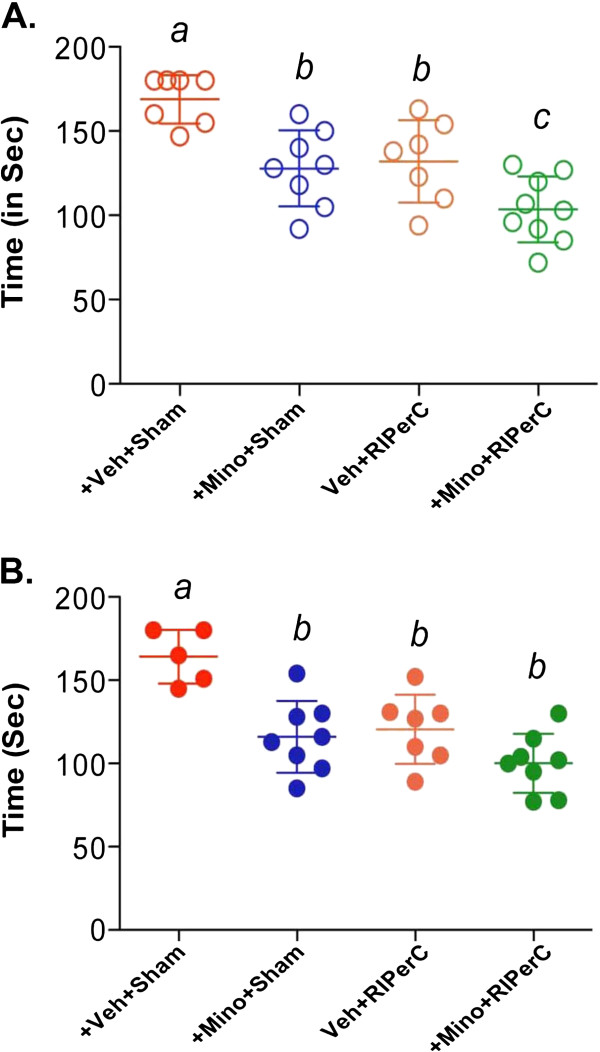
**Sensorimotor function test by adhesive tape removal at 48 hrs post-stroke. A**. Experiment I without IV-tPA, and **B**. Experiment II with IV-tPA. Data presented as Mean ± SD (N = as above in Figure 1). Pairs of Means with different letters are significantly different, p < 0.05.

We further tested surviving mice for sensorimotor function outcomes (Figures 3A-B) at 48 hrs post-stroke. In Experiment I without thrombolysis, there was no interaction between MINO and RIPerC (F_(1,27)_ = 0.71, p = 0.4073) but individual treatments with MINO (F_(1,27)_ = 21.73, p < 0.0001) and RIPerC (F_(1,27)_ = 16.87, p = 0.0003) significantly improved sensorimotor function as compared to the sham-treatment control without thrombolysis. Interestingly, while the outcome remains similar to individual treatments (sham-operated control vs. MINO only p = 0.0035, sham-operated control vs. RIPerC only p = 0.0122) in the NDS test, the combination therapy showed improvement in the sensorimotor function outcomes (p = <0.0001 vs. sham-treated without thrombolysis group; and p = 0.0499 vs. RIPerC alone). In the Experiment II with thrombolysis, there was no interaction between MINO and RIPerC (F_(1,24)_ = 3.45, p = 0.0756) but individual treatments with MINO (F_(1,24)_ = 21.10, p = 0.0001) and RIPerC (F_(1,24)_ = 15.86, p = 0.0006) significantly improved sensorimotor function as compared to the sham-treatment control. MINO (p = 0.0011), RIPerC (p = 0.0041) and their combination (p < 0.0001) improved the sensorimotor functions significantly but there was no additional benefit on sensorimotor function outcome after combination therapy as compared to individual MINO and RIPerC treatments.

### MINO and RIPerC individual treatments and their combination after embolic stroke reduced lesion size with and without IV-tPA

**Figure 4:**
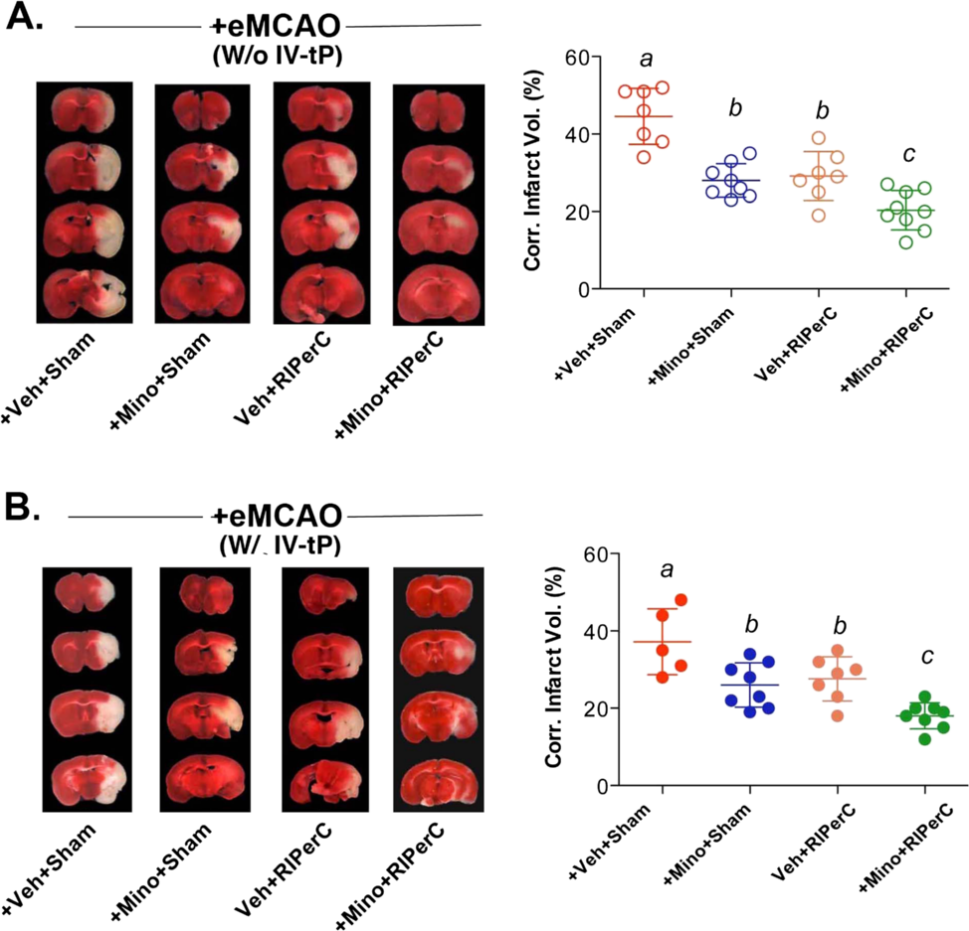
**Representative TTC stained coronal sections, and Means of the corrected infarct volumes calculated as percent of their corresponding contralateral sides. A**. Experiment I without IV-tPA, and **B**. Experiment II with IV-tPA. Data are presented as Mean ± SD (N = as above in Figure 1). Pairs of Means with different letters are significantly different, p < 0.05.

In Experiment I without thrombolysis, there was no interaction between MINO and RIPerC (F_(1,27)_ = 3.48, p = 0.0728) but the MINO (F_(1,27)_ = 37.26, p < 0.0001) and RIPerC (F_(1,27)_ = 30.85, p < 0.0001) individual treatments significantly reduced the lesion size as compared to the sham-treatment control (Figure 4A-B). Moreover, the combination of MINO and RIPerC further decreased the injury size as compared to the sham-treatment control (p < 0.0001) as well as both individual treatments (MINO alone p = 0.0495, RIPerC alone p = 0.0254), and each individual treatment (MINO alone p < 0.0001, RIPerC alone p = 0.0002) decreased injury size as compared to the sham-treatment control. In Experiment II with thrombolysis, the lesion outcome remained similar to Experiment I where there was no interaction between MINO and RIPerC (F_(1,24)_ = 0.13, p = 0.7171) but the MINO (F_(1,24)_ = 21.87, p < 0.0001) and RIPerC (F_(1,24)_ = 15.75, p = 0.0006) individual treatments reduced injury significantly as compared to the sham-treated group. The combination treatments showed an additive effect, which was significantly different in reducing the injury size as compared to the sham-treated control (p < 0.0001) as well as individual treatments (MINO alone p = 0.0484, RIPerC alone p = 0.0185). Each individual treatment (MINO alone p = 0.0116, RIPerC alone p = 0.0410) decreased injury size as compared to the sham-treated control.

## Discussion

In our physiological thromboembolic stroke model we used “late IV-tPA” at 4 hours to model the “end of the therapeutic time window” in humans where IV-tPA often does not benefit. Our data demonstrate that both MINO alone and RIPerC alone reduce infarct size and improve short-term functional outcomes in mid aged mice either treated or not treated with IV-tPA. In addition, the combination treatment of RIPerC and MINO in mice reduced infarct size significantly compared to either intervention alone in both settings of with or without thrombolysis by IV-tPA. We could not demonstrate a significant improvement in functional outcomes with the combination compared to either intervention alone. However, the combination did improve sensorimotor function as measured by the adhesive tape removal test in comparison to either agent alone in mice that did not receive IV-tPA. This lack of additional benefit with the combination in the other groups may be due to a “ceiling effect” and the lack of sensitivity of these tests in mice. Overall, the combination of MINO and RIPerC reduces infarct size and there are trends in favor of functional improvement compared to either treatment alone.

Importantly, we did not find a statistical “interaction” between MINO and RIPerC in our 2 × 2 factorial design for all outcomes without IV-tPA (see Additional file 1: Figure S1a for an example) and all outcomes except NDS with IV-tPA. A statistical interaction would indicate that there was a synergistic effect of MINO and RIPerC rather than an additive effect such that the combination would increase or decrease the mean more than anticipated if the effect due to MINO alone and the effect due to RIC alone were added together. While there was an interaction between MINO and RIPerC in NDS testing in the IV-tPA treated animals (Additional file 1: Figure S1b), there were no other interactions found in the other outcome measures, i.e., cerebral perfusion, sensorimotor function outcome, and infarct size, in the IV-tPA and without IV-tPA treated mice. In a 2 × 2 factorial design for a clinical trial, the knowledge of potential interactions between the agents, influences samples size estimation [16,17]. If there is no significant interaction, the sample size for the trial is smaller and “two agents” can be tested efficiently and economically, “ two trials for the price of one”.

RIPerC triggers endogenous protective pathways and works by multiple mechanisms that include improvement of CBF [18]. MINO also has multiple mechanisms of action that includes inhibition of MMP-9 [11,19,20], PARP-1 [20], and peroxynitrite [21], and also possesses anti-inflammatory actions via inhibition of microglial activation [22,23]. The STAIR VII recommended that stroke therapy “focus on drugs/devices/treatments with multiple mechanisms of action and that target multiple pathways” [1]. Both of our interventions target multiple mechanisms and cascades triggered by ischemia. RIPerC induces a “protective cerebral phenotype” and has the added benefit of increasing CBF.

### Limitations of the study

Some limitations of our study are that we only focused on short-term outcome, used only male animals and we did not study animals with comorbidities such as hypertension or diabetes. However, we did study in mid aged mice, as age is an important comorbidity. In other studies, we have shown gender independent effects with both MINO and RIPerC [13,15]. We also administered just one dose of MINO and performed one time RIPerC-therapy after stroke. Repeated doses of MINO and repeated conditioning regimen may have provided even more robust protective effects.

The recent Danish pre-hospital stroke trial of RIPerC in the ambulance showed feasibility, safety and tolerability of RIPerC [6]. While the primary outcome, penumbral salvage, was neutral, there was evidence of tissue protection in a post hoc tissue survival analysis. The baseline hospital National Institutes of Health Stroke Scale Score (NIHSS) in this trial was low (median 5), indicating that the strokes were very mild. Moreover, the majority of the patients did not receive the full conditioning regimen of 4 cycles of 5 minutes occlusion. Enrolling more severe strokes and ensuring that the conditioning regimen is completed and even repeated will be important in future clinical trials.

## Conclusion

In order to move the acute stroke therapy forward, innovative delivery systems, therapies and clinical trials designs are needed. This pre-clinical 2 × 2 design can be easily translated into a pre-hospital clinical trial. With the increased use of IV-tPA, the extended time window of IV-tPA out to 4.5 hours, and the increased availability and the expanded use of mechanical reperfusion, it is imperative that safe agents and interventions that can “freeze” the penumbra be tested and developed. The combination of these two feasible, safe and inexpensive interventions to treat acute stroke in the ambulance has potential high impact and huge cost savings, and therefore, should be tested in clinical trial.

## Competing interests

The authors declare that they have no competing interests.

## Authors’ contributions

MNH designed the study; carried out the stroke surgery and laser speckle contrast imaging (LSCI); helped in the data analysis and interpretation; helped to draft and revise the manuscript. SCF helped to draft the manuscript and made critical revision of the manuscript; handled funding and supervision. MBK helped in treatments, post-surgery care, and helped in the data analysis and interpretation. KV handled the animal colony, and helped in the coordination of study, post-surgery care, animal sacrifice and tissue collection, and data analysis and interpretation. AC assisted during the surgery, helped in the calculations and data analysis. PW helped in animal procurement, coordination of behavioral study and helped to revise the manuscript; handled funding and supervision. KMD helped in coordination of behavioral study and revision of the manuscript; handled funding and supervision. JLW performed the statistical analysis; helped in the manuscript writing and critical revision. DCH conceived and designed the study; helped in the study coordination; analyzed and interpreted the data; helped to draft the manuscript and made critical revision of the manuscript; handled funding and supervision. All authors read and approved the final manuscript.

## Supplementary Material

Additional file 1: Figure S1 Supplementary figure showing **(a)** No statistical interaction for Neurological Deficit Score (NDS) without IV-tPA in Experimental Set I, while **(b)** there is a statistical interaction in the Experimental Set II performed with IV-tPA at 4 hrs post stroke. Click here for file
