# Supplementary figures and images for: A 2 × 2 factorial design for the combination therapy of minocycline and remote ischemic perconditioning: efficacy in a preclinical trial in murine thromboembolic stroke model
Source: Exp Transl Stroke Med. 2014 Oct 9;6:10. doi: 10.1186/2040-7378-6-10 (PMC4204390; doi:10.1186/2040-7378-6-10)

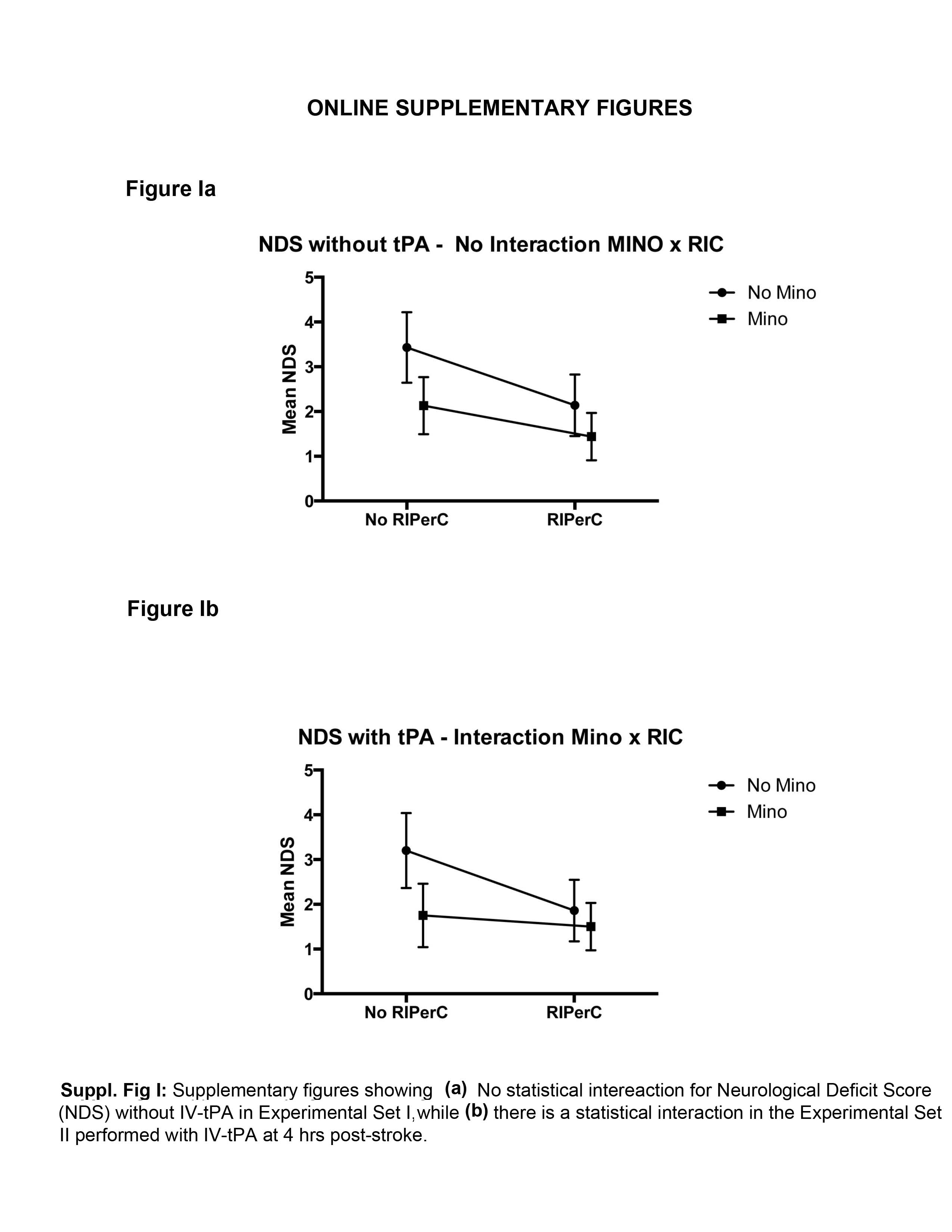

Supplement: Additional file 1: Figure S1 — Supplementary figure showing (a) No statistical interaction for Neurological Deficit Score (NDS) without IV-tPA in Experimental Set I, while (b) there is a statistical interaction in the Experimental Set II performed with IV-tPA at 4 hrs post stroke. [file 2040-7378-6-10-S1.jpeg]
